# Case Report: Projectile Into Right Frontal Lobe From a Nail Gun

**DOI:** 10.7759/cureus.9460

**Published:** 2020-07-29

**Authors:** Tye Patchana, Taha M Taka, Hammad Ghanchi, James Wiginton, Margaret Wacker

**Affiliations:** 1 Neurosurgery, Riverside University Health System Medical Center, Moreno Valley, USA; 2 Neurosurgery, University of California Riverside, Riverside, USA; 3 Neurosurgery, Arrowhead Regional Medical Center, Colton, USA

**Keywords:** nail gun, brain projectile, projectile, work place trauma

## Abstract

We present a case of a nail gun injury penetrating the right maxillary sinus and frontal lobe with complaints of headache and right eye blindness. After surgical removal and treatment, there were no neurological deficits aside from the persistence of right eye blindness that the patient initially presented with. Our report describes the patient’s clinical course, the multidisciplinary medical and surgical management, along with the clinical workup and important mental health considerations for patients presenting with intracranial nail gun injuries.

## Introduction

Nail gun injuries are a common workplace accident causing 37,000 emergency room visits annually [1]. While a vast majority of these injuries are to the hands and fingers, a small fraction of nail gun injuries occur to the cranium, which poses a significant challenge for neurosurgeons. Per the Centers for Disease Control and Prevention (CDC) data on nail gun injuries, those involving the intracranial cavity account for a small subset - approximately 0.1%, of all nail gun injuries [2]. These patients are typically males in their second decade of life. As nail guns become more powerful and available to the public, the frequency of these injuries is increasing [3]. However, the low prevalence of intracranial nail gun injuries from accidental discharge as compared to overall nail gun injuries should alert physicians to keep non-accidental discharge in mind during clinical workup. A review of Amazon’s offerings of nail guns shows that these products may be obtained for as little as $29.99. Most nail guns operate at up to 100 pounds per square inch (PSI). Some of the most powerful nail guns operate at pressures 100-130 PSI. A previous study investigating high-pressure injection injuries to cadaver human hands determined penetration of tissue results in little trauma laterally with most of the damage occurring in the path of penetration [4].

Intracranial nail gun injuries carry a more favorable prognosis than projectile trauma secondary to traditional firearms, likely due to their lower relative velocity [5]. A review of the literature shows that most cases carry a good prognosis for the patient, although reports of neurological deficit and death have also occurred [6,7]. Pseudoaneurysm formation is the most significant concern following injury. These lesions may develop in a delayed fashion (up to three weeks after injury), and necessitate cerebral angiography following injury [3]. Other concerns include the formation of hemorrhage. It is important to note that an artifactual scattering effect from the nail may obscure hemorrhage on imaging studies. We present this case of a nail gun injury to the right maxillary sinus and frontal lobe in order to highlight the clinical course, surgical management and planning, and several important clinical considerations.

## Case presentation

**Figure 1:**
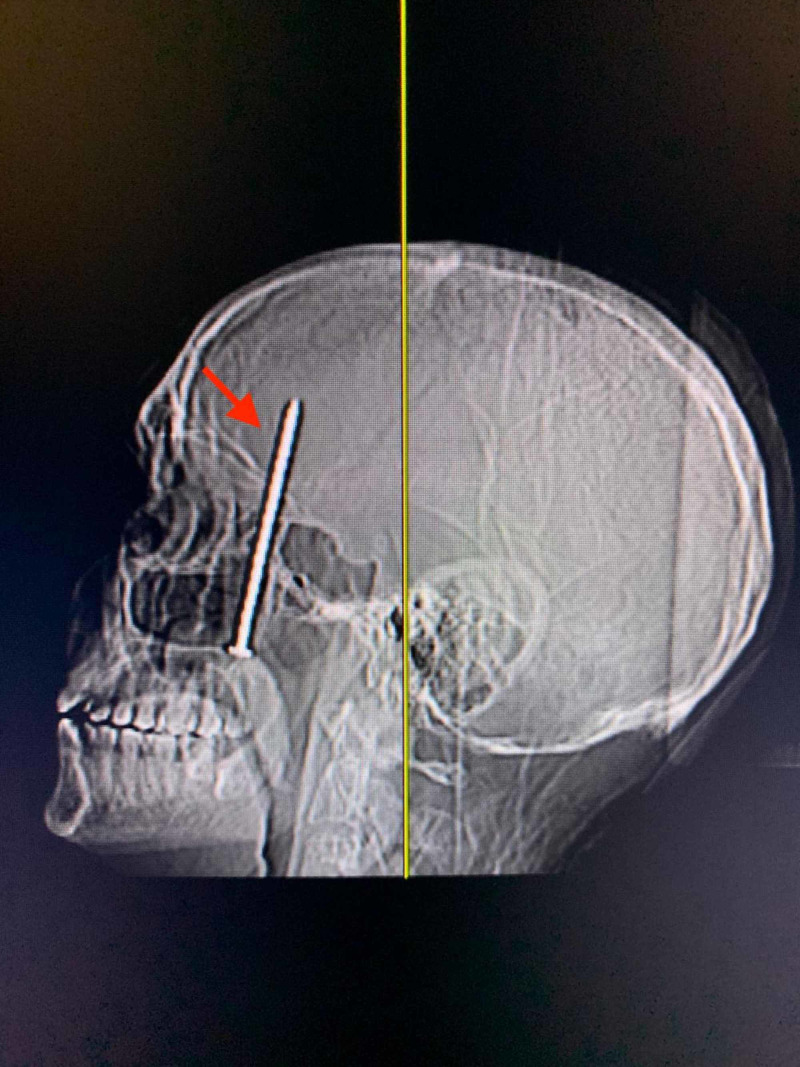
Lateral X-ray of the skull demonstrating penetration of the anterior skull base

**Figure 2:**
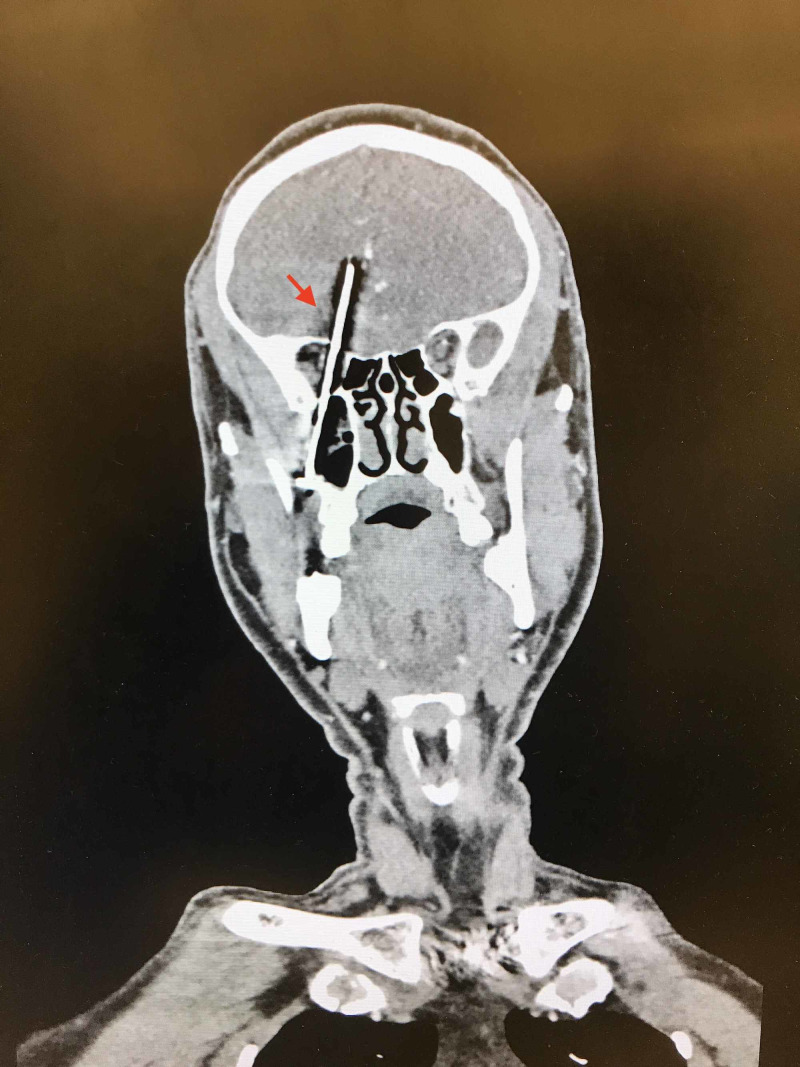
Coronal CT demonstrating penetration of the anterior skull base, demonstrating proximity to the anterior cerebral artery (ACA)

The patient is a 50-year-old Hispanic male presenting to the ED with chief complaints of headache and right eye blindness. The patient had been working construction with a nail gun that had reportedly misfired resulting in a foreign body projectile into the calvarium. While the patient did not report losing consciousness, he was unaware of the misfire and did not know the reason for his new symptoms. X-ray of the skull and CT scan of the head demonstrated a foreign object resembling a nail extending through the right maxillary sinus into the right anterior skull base, penetrating the frontal lobe of the brain (Figure 1). A CT Angiogram of the head was negative for any vascular abnormalities, therefore a diagnostic cerebral angiogram was deferred. The patient denied any neurological deficits with the exception of right eye blindness. No signs of cerebrospinal fluid (CSF) leak were present from the nose or into the mouth. A Maxillofacial CT demonstrated the nail passing through the right maxillary sinus, penetrating the floor of the orbit, but posterior to the globe. The trajectory of the nail was through the base of the skull, protruding significantly into the brain parenchyma. The tip of the nail was located adjacent to the right anterior cerebral artery (Figure 2). Surgical removal was indicated due to the risk of CSF leak, nidus of infection, and potential further intracranial injury. The location of the head of the nail within the oral cavity necessitated the coordination of Neurosurgery with Oral Maxillofacial Surgery (OMFS) for a multidisciplinary approach for the removal of the foreign body. Preoperatively, the patient was provided with antibiotic coverage.

**Figure 3:**
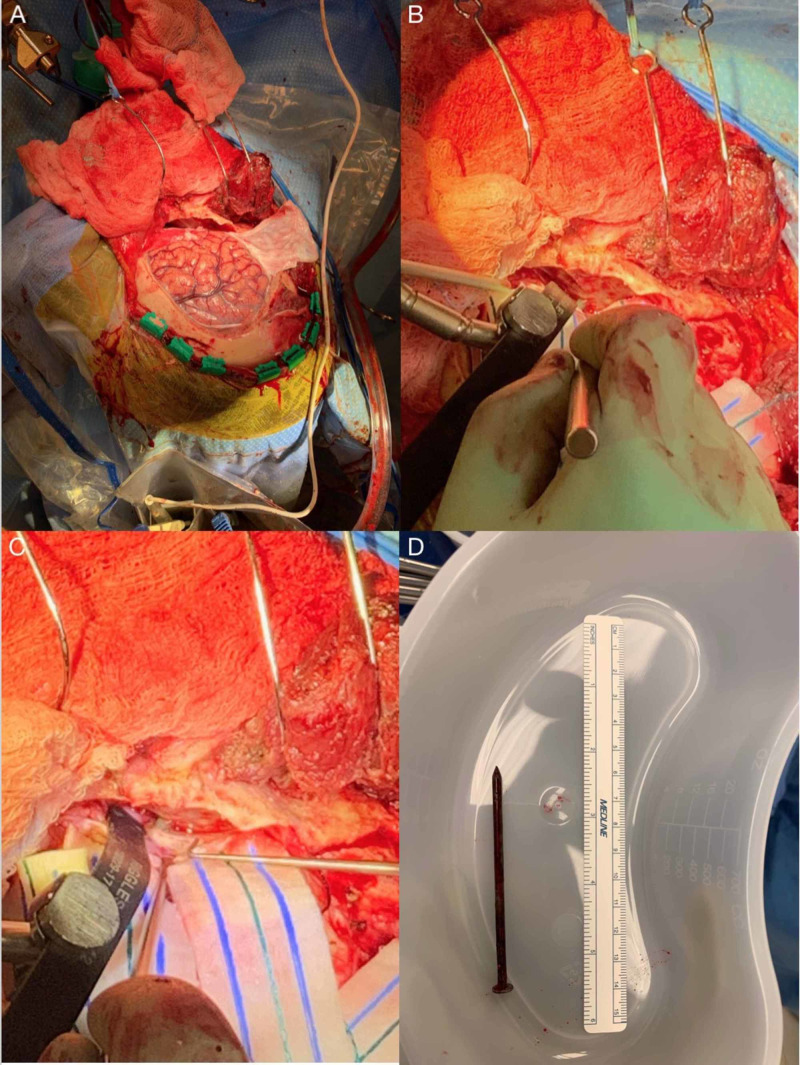
(A) A craniotomy was performed with further dissection continued anteriorly down to the dura. (B) Brain ribbon to retract the frontal lobe away from the skull. (C) A pericranial flap was placed over the dural defect, overlaid by a piece of DuraGen, and DuraSeal was applied extradurally. (D) Approximately 10 cm nail removed status post craniotomy

A craniotomy was performed with extradural dissection continued anteriorly down the posterior orbital wall until the calvarial entry point of the nail was appreciated. The dura was dissected around the nail using a brain ribbon to retract the frontal lobe away from the skull. At this time, OMFS removed the nail through the oral cavity. Following removal, meticulous hemostasis was achieved. Ultrasound was used to confirm the absence of parenchymal hematoma after the removal of the nail. A pericranial flap was placed beneath the dural defect, overlayed by a piece of DuraGen™, and DuraSeal™ was applied extradurally to ensure against CSF leak (Figure 3).

The patient continued to do well throughout the postoperative course, and was discharged after a period of observation. The patient presented to the clinic several weeks later with continued blindness in his right eye.

## Discussion

Treatment of cranial foreign object penetration focuses on the removal of the object while avoiding further secondary damage from vascular malformation and infection. For this reason, pre-operative evaluation should include thin-cut CT scans and assessment of vasculature by angiography [1,2] in cases where the nail appears to traverse any major vessels. It is important to note that the artifactual scattering effect of the nail may hinder efforts to detect nearby hemorrhage.

Additionally, due to the neurological injury that may be caused secondary to removal of the object, surgical management of intracranial nail may require an emergent craniotomy. Post-operative imaging and clinical observation for at least 24 hours are recommended to visualize any pseudoaneurysm formation or to detect any secondary neurological injury. Additionally, the repetition of angiography at 7-14 days post-injury is prudent as nail gun injuries have a high risk of pseudoaneurysm formation [3].

The increasing use of nail guns with the sequential-trip trigger safety mechanism, which requires the head of the nail gun to be pressed prior to pressing the trigger for the nail protrusion, has decreased the likelihood of accidental nail gun injuries. This fact, along with the previous studies reporting a high occurrence of non-accidental nail gun injuries should clue physicians into the possible need for psychiatrist involvement [3,8]. Depending on the nature and location of the trauma, surgical and medical treatment of nail gun injuries may require the collaborative effort of ophthalmology, otolaryngology, oral maxillofacial surgery, and psychiatry.

## Conclusions

We present a case of a 50-year-old man presenting to the ED with right eye blindness and headache secondary to accidental nail gun discharge, leading to penetration of the right maxillary sinus, anterior skull base, and brain parenchyma. The patient underwent a craniotomy to uncover the nail’s entry point and subsequent removal of the nail from the oral cavity while monitoring for CSF leak. This case highlights the necessary screening and surgical efforts taken to ensure the absence of a pseudoaneurysm and further damage secondary to projectile removal. Additionally, we highlight the importance of a high level of suspicion for non-accidental nail gun injuries.
